# A geolocated dataset of German news articles

**DOI:** 10.1038/s41597-025-05422-w

**Published:** 2025-07-02

**Authors:** Lukas Kriesch, Sebastian Losacker

**Affiliations:** 1 Department of Geography, Justus Liebig University Giessen, Senckenbergstr. 1, 35390 Giessen, Germany; 2 CIRCLE—Center for Innovation Research, Lund University, Lund, Sweden

**Keywords:** Geography, Society, Economics

## Abstract

The emergence of large language models and the exponential growth of digitized text data have revolutionized research methodologies across a broad range of social sciences. News data is crucial for the social sciences as it provides real-time insights into public discourse and societal trends. In this paper, we provide insights into how news articles can be geolocated and how the texts can then be further analyzed. We collect data from the CommonCrawl News dataset and clean the text data. We then use a named-entity recognition model for geocoding. Finally, we transform the news articles into text embeddings using SBERT, enabling semantic searches within the news data corpus. In the paper, we apply this process to all German news articles and make the German location data, as well as the embeddings, available for download. We compile a dataset containing text embeddings for about 50 million German news articles, of which about 70% include geographic locations. The process can be replicated for news data from other countries.

## Background & Summary

The emergence of large language models (LLMs) and the exponential growth of digitized text data have revolutionized research methodologies across the social sciences^1–3^. The wealth of available digital sources has equipped researchers with the ability to conduct systematic, large-scale analyses through natural language processing (NLP) techniques. Text-based indicators offer a valuable complement to traditional metrics, capturing timely, topic-specific, and regionally differentiated patterns in public discourse, policy debates, and social dynamics^4–6^. The power of these indicators lies in their ability to reveal emerging trends, capture societal reactions to new developments, and highlight early signs of policy adoption or resistance, among many other fields of application. In geographical research, text-based analysis provides new insights into various phenomena at the local level, where established data sources often fall short. It helps to capture local and regional dynamics that are often overlooked by traditional macro-level indicators. Recent studies have explored novel text-based data sources, including geolocated firm web pages, for regional analyses, but other types of geolocated text data have remained underutilised, leaving the rich potential of text data unexploited^7–10^.

While corporate and economic data provide structured indicators, they often miss the broader cultural and social narratives that drive public opinion and collective behavior on the regional level. Researchers interested in studying social science phenomena from a geographical perspective therefore rely on additional data sources.

This is where news data becomes crucial: news articles embody the sentiments, perspectives, and legitimacy processes that underpin how events, policies, innovations, and societal changes are discussed, debated, and integrated into everyday life^11–13^. Such an “outside-in” approach offers researchers a window into public perception and the underlying forces shaping societal changes across various regions.

Applications of news data in the social sciences have contributed to fields such as economics^14–17^ and political science^18,19^. Several prominent datasets have demonstrated the value of geographically disaggregated text-based data for social science, especially in the domains of conflict monitoring, crisis forecasting, and international relations^20,21^. Complementing event-based datasets, full-text news corpora hold great promise for producing qualitative, regionally specific indicators^22–25^. They serve as a rich repository of local narratives, offering information on how regional identities, subnational dynamics, and cultural variations influence public acceptance and discourse around policies, innovations, and social movements, among other aspects.

In this paper, we present a comprehensive approach to analyzing large-scale news data by leveraging pre-trained transformer models, known for their exceptional semantic understanding. Focusing on the German subset of a global news corpus, we harness these capabilities to process and index extensive text data, enabling detailed insights into regional and thematic trends within German news coverage. Key to this methodology is the combination of data retrieval, advanced text embeddings, and semantic search, allowing for precise extraction and categorization of articles. Although the dataset described in this paper is limited to German news articles due to computational constraints and the feasibility of manual quality control, our approach can be adapted and replicated for processing news articles from other countries and in other languages. The paper’s structure is designed to guide readers through the data processing workflow, from acquisition to practical applications. The robustness of this approach is verified through both qualitative analysis and quantitative metrics, emphasizing the model’s adaptability and efficacy. The final dataset published with this article consists of an SQLite database and a Usearch vector database, which together provide comprehensive data storage and semantic search capabilities. The SQLite database contains structured information about news articles and their associated geographic locations, while the Usearch vector database enables efficient semantic search through vector representations of the articles.

## Methods

**Fig. 1:**
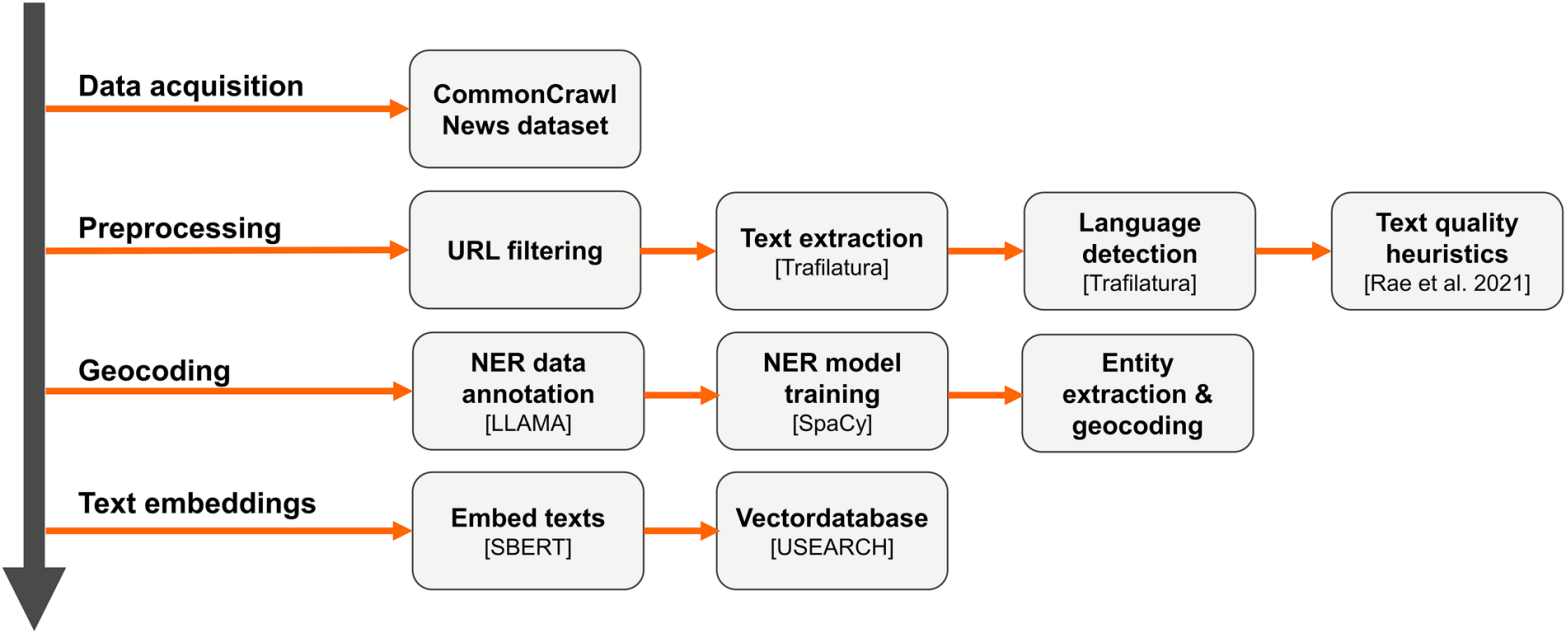
Data processing pipeline.

Figure 1 illustrates the end-to-end processing pipeline for transforming raw web data into a structured dataset suitable for analysis. The process begins with downloading the dataset, followed by filtering out webpages with non-German country-specific top-level domains (TLD) to focus on relevant content. Afterwards, we extracted the primary text content from the raw HTML and retained only articles identified as German through language detection. Low-quality content was filtered out using established text quality heuristics^26^.

Next, we prepared training data for a custom named-entity recognition (NER) model to identify relevant entities. This model was used for comprehensive entity extraction and geocoding, linking entities to specific geographic locations. Finally, each news article text was transformed into a text embedding using SBERT, and these embeddings were stored in a vector database, allowing for efficient semantic search and downstream analysis. Detailed descriptions of each processing step are provided below.

### Stage 1: Data acquisition

For data acquisition, we utilize the Common Crawl News dataset, a resource curated by the non-profit organization Common Crawl, which has been systematically crawling the web since 2007. This organization releases new collections of web content at intervals of 1 to 2 months. Since August 2016, Common Crawl has maintained a dedicated news dataset, using RSS/Atom feeds and news sitemaps to discover and aggregate links to articles across a wide spectrum of news platforms.

The dataset is provided in the WARC (Web ARChive) format, which includes both the complete HTML content of each webpage and metadata from the HTTP requests. This archival format enables researchers to access comprehensive records of web pages as they appeared at the time of crawling, which is essential for robust historical analysis and reproducibility in research.

**Fig. 2:**
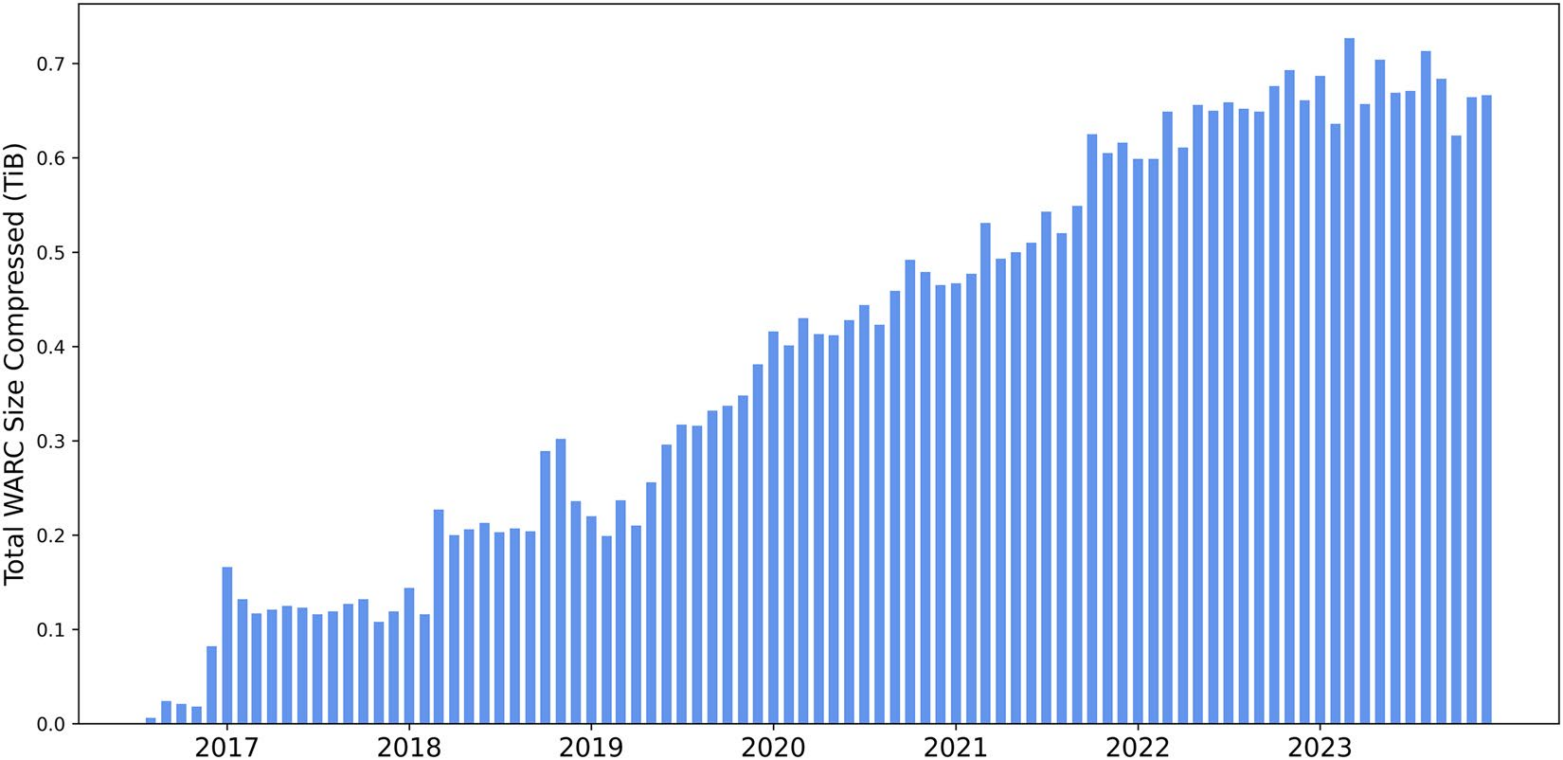
Size of news data collected by Common Crawl over time.

Figure 2 depicts the size of each news crawl from August 2016 to December 2023 showing a consistent increase in the total compressed size of WARC files over this period. This growth trend highlights the substantial expansion of archived web data, with particularly rapid increases observed during and after 2020. The dataset used for this study encompasses over 35 TiB of uncompressed HTML text content, representing a vast source of raw news data. Access to this data is facilitated through Amazon S3 buckets or by direct download from the Common Crawl servers, allowing flexibility for both cloud-based and local data processing.

### Stage 2: Text extraction and pre-processing

The second stage of the pipeline involves filtering, extracting and preparing the raw HTML content from the Common Crawl News dataset for downstream analysis. Given the dataset’s scale and heterogeneous sources, this stage is critical for transforming unstructured web data into clean, structured text suitable for text analysis and machine learning tasks.

In a first step, we removed entries with non-German country-specific TLD. This targeted filtering reduces the dataset’s volume by eliminating sources that are unlikely to provide German-language content, thereby streamlining the subsequent language detection and quality filtering processes.

To ensure the relevance and quality of the extracted content, we eliminated extraneous elements such as navigation menus, headers, footers, boilerplate text, and advertisements. We employed the Trafilatura library for this purpose, which efficiently extracts not only the main body of the article but also associated metadata including titles, tags, categories, and excerpts when available^27^. Additionally, we utilized Trafilatura’s built-in language detection algorithm to extract only German-language texts. Trafilatura has been validated as a fast and reliable tool for text extraction from web pages, offering significant improvements in text quality and accuracy^28,29^.

Web text data often contains substantial amounts of low-quality or poorly formatted content, which can introduce noise and diminish the accuracy of subsequent analyses. To mitigate this issue, we applied a rigorous filtering process based on established text quality heuristics^26,30^:

1. We removed articles with five or fewer sentences to eliminate content that might lack depth and context, often appearing as stubs or summaries.
2. Articles with more than 10% non-alphabetic words were also filtered out, as this could indicate a prevalence of numbers, symbols, or code snippets instead of narrative text.
3. We excluded articles averaging five or fewer words per line to avoid lists, tables, or poorly formatted content that does not resemble standard prose.
4. We excluded articles containing JavaScript code.
5. We retained articles with an average word length between 3 and 10 characters to ensure the language is typical, avoiding overly technical jargon or overly simplistic words.
6. We removed duplicate articles by retaining only unique combinations of text and news provider.
7. We kept articles with a word count between 50 and 10,000 to exclude those too short to be informative and those excessively long, which might not represent genuine news content.

This approach enabled us to systematically identify and exclude substandard data, ensuring that our corpus consisted of high-quality, reliable texts. Table 1 depicts the number of articles removed per criterion.

**Table 1:** Summary statistics of removed articles.

Criterion	Removed articles count	Share of dataset	Share of removed articles
Fraction non alphanumeric words ≥0.1	570,423	1.14%	86.86%
Word count ≤50	83,751	0.17%	12.75%
words_per_line ≤5	50,655	0.1%	7.71%
sentences_count <3	46,808	0.09%	7.12%
javascript_count >0	8,056	0.01%	1.22%
word_count ≥10000	1,017	0.00%	0.15%
mean_word_length >12	304	0.00%	0.005%
mean_word_length <3	48	0.00%	0.007%

**Fig. 3:**
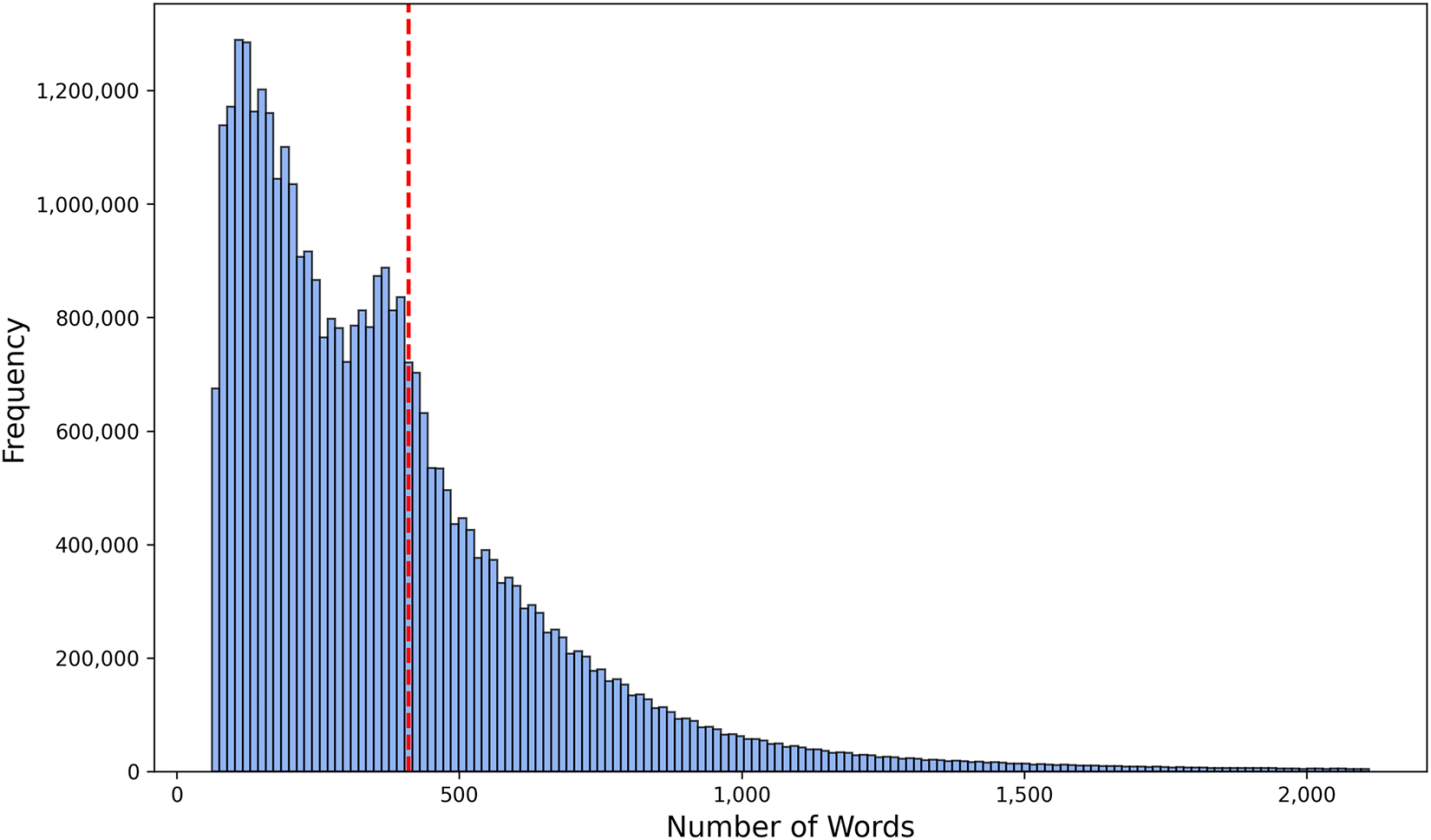
Word count per article (top and bottom 1% excluded); mean article length is marked by the red-dashed line.

After completing these preprocessing steps, the dataset comprises 49,374,999 German news articles. The articles are predominantly full text, averaging 410 words, as shown by the red dashed line in Fig. 3. Relying on full text, rather than just headlines, is critical to grasping the depth and complexity of each article’s narrative. While headlines are effective at capturing attention, they often prioritise engagement over nuance and can potentially skew the underlying message unintentionally. By analysing full texts, this dataset ensures a more robust representation of news narratives and sentiments, capturing subtle shifts in discourse that might otherwise be overlooked.

### Stage 3: Named entity recognition

The geocoding of news articles is an important step for geographical analyses of news, and there are different ways in which a news article can relate to a specific geographical area or place. When analyzing news articles from a spatial perspective, it is essential to recognize that news is more than a mere collection of isolated events. News is created by individuals (I) to inform others (II) about events occurring in specific locations (III)^22^. (I) The location of production represents where journalists and editors craft the content, potentially shaping how stories are framed and selected. (II) The consumption location reflects where the news is consumed, which can shape local opinions and decision-making. (III) Lastly, the event location indicates the geographic context of the incidents being reported, offering collective insights and perceptions of what is happening in a given area or place.

These spatial dimensions—production, event, and consumption—are critical for understanding how news circulates across regions and how it influences sentiments and perceptions in different areas. While detailed data on readership is often scarce, we focus in this paper on the relationship between locations and events, aiming to understand how regional narratives and discussions around specific topics develop and vary. To effectively analyze these spatial dimensions, within news articles, it is essential to accurately identify and extract geographic entities. We employed a multi-step NLP pipeline to extract location entities from a large corpus of German news articles. Initially, we utilized Meta’s LLAMA-3.1-8B-Instruct model for generating entity extraction responses. To process the text data, we sampled and deduplicated articles, yielding a set of 50,000 unique texts for analysis. Each text was inputted into the model along with a predefined system prompt designed to elicit structured JSON responses containing identified location entities. To ensure that the extracted entities met the expected types we validated them using Pydantic models^31^. Following validation, the entities were incorporated into spaCy’s processing pipeline to train a custom Named Entity Recognition (NER) model specifically designed to identify city names within the news articles. The use of place names as point data enables precise assignment to territorial and statistical units. As a result, federal state and district names are excluded. Similarly, landmarks, street names, and square names are omitted, as their clear allocation cannot be guaranteed.

We chose spaCy for its fast CPU inference and lightweight model architecture, which is ideal for deploying efficient NER systems at scale. The performance metrics of this custom NER model are detailed in the technical validation section.

Using the LLM for annotation provided substantial benefits in scalability and consistency, as manually annotating such a large dataset would have been impractically time-consuming and resource-intensive. We considered the potential for biases in the LLM’s annotations, which could reflect its training data. Notably, since the Common Crawl dataset is part of the LLM’s training corpus, we infer that the model has robust knowledge of named entities in news articles. Manual inspections of the training data did not reveal any significant biases.

Despite these limitations, the annotations provided by the LLM served as high-quality training data for our custom NER model. This model achieved strong performance metrics and significantly reduced inference time, a crucial factor for handling large datasets. We utilized the model to extract location entities from the entire database of articles.

### Stage 4: Geoparsing

Having extracted candidate location mentions using our NER model (Stage 3), we now describe the process of resolving these text-based entities to geographic coordinates.

Accurately mapping location names from news articles to geographic coordinates is a challenging task due to name ambiguity and the presence of multiple variants for the same place (e.g., “Frankfurt” vs. “Frankfurt am Main”). To address this, we implemented a geoparsing pipeline that combines named entity recognition (NER), entity normalization, and geocoding with multiple validation steps. First, we enriched our NER training data with fully qualified toponyms (e.g., Frankfurt am Main, Halle an der Saale) as well as their common abbreviations and orthographic variants (e.g., Frankfurt/Main, Frankfurt a.M.). This targeted annotation enabled the model to better normalize and distinguish location mentions before geocoding. After extraction, all entities were standardized by lowercasing, removing special characters, and trimming whitespace. To reduce noise, we filtered out rare mentions (fewer than 100 occurrences). Geocoding was performed using the Nominatim API, which queries the OpenStreetMap (OSM) gazetteer. By default, Nominatim returns the most “important” (typically the most populous or well-known) match first^32^. This default behavior resolved many duplicate-name conflicts using a simple heuristic by prioritizing the most populous or prominent location associated with a given location name. Each matched location was assigned to a German NUTS-3 region. While our approach does not leverage document-level context as some more advanced geoparsers do, it offers a simple and scalable solution for large-scale geoparsing^33,34^. However, several limitations remain. First, our approach does not incorporate document-level contextual information that could help disambiguate locations with identical names. Second, the reliance on Nominatim introduces potential biases due to how OpenStreetMap ranks and matches locations—favoring more populous or internationally prominent cities. Third, abbreviated or informal place name variants may not always resolve correctly if not explicitly included in our training data. We provide the raw geoparsing results with latitude, longitude, and original text mention, enabling users to apply their own disambiguation strategies as needed. Moreover, we conducted several validations to assess the accuracy of our geoparsing pipeline, which are detailed in the technical validation section. Through our pipeline, we successfully resolved at least one valid city name in 36,305,239 articles, i.e., approximately 70% of the entire corpus contained at least one valid city name.

### Stage 5: Embedding transformation

To facilitate semantic analysis of the geocoded data, we embedded the news articles using sentence transformers, converting the text data into numerical vectors. We encoded the articles with a “passage: “ prompt, ensuring that the model treats each article as a discrete textual passage. This approach prepares the passages to be found effectively via semantic search, enhancing retrieval relevance and specificity for nuanced content queries. We employed the “deepset-mxbai-embed-de-large-v1” model to embed the texts^35^. This model facilitates Matryoshka Representation Learning and Vector Quantization, which effectively reduces memory consumption when analysing the data at scale. Additionally, we offer the embeddings in different vector dimensions and precisions, enabling semantic search at varying levels of precision and compatibility with diverse hardware requirements. We detail the usage of the vector database for article search and retrieval in the data records section.

## Data description

This section provides an overview of the geolocated dataset. The complete published dataset includes all articles, including those without linked geographic information.

**Fig. 4:**
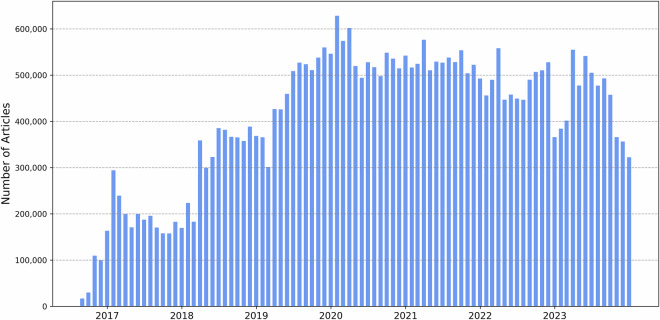
Number of German news articles per month.

Figure 4 illustrates the temporal distribution of news coverage per month, revealing a steady increase in the number of articles since the commencement of crawling in August 2016, with a peak reached in early 2020 during the COVID-19 outbreak in Germany. Since then, the number of articles per month has stabilized at around 500,000, with only minor seasonal fluctuations.

**Fig. 5:**
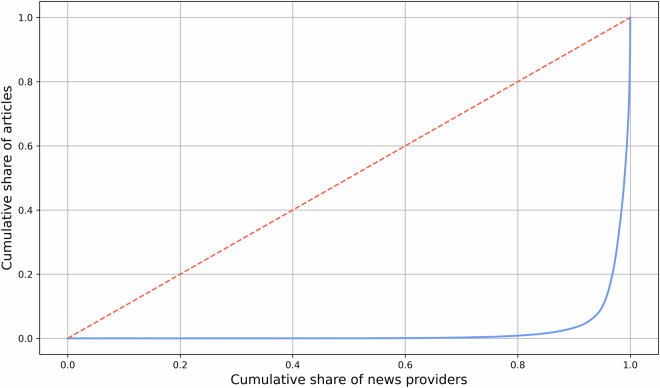
Lorenz curve of the distribution of German news articles across domains.

The database encompasses news items from 3,211 different domains. The Gini coefficient of 0.95 for the distribution of news articles across domains in Germany underscores a significant concentration of news media production, with a few large providers dominating the landscape. Figure 5 displays the lorenz curve of the distribution.

This high level of skewness indicates that, although numerous media outlets exist, the majority of news content is generated by a small number of major players. This concentration results from the market dominance of these major outlets, which possess the resources and infrastructure necessary to produce and distribute news on a larger scale. However, the dataset also includes news from many smaller outlets, ensuring a diverse range of perspectives and regional coverage. This diversity is crucial for capturing the nuances and variations in news reporting across different regions.

## Data Records

The data is stored in a repository hosted by the University of Giessen^36^. The dataset consists of an SQLite database and a Usearch vector database, which together provide comprehensive data storage and semantic search capabilities. The SQLite database contains structured information about articles and their associated geographic locations, while the vector database enables efficient semantic search through vector representations of the articles. The article titles, texts and excerpts associated with this data can be retrieved directly from Common Crawl and linked to this dataset using the provided IDs. The table schemas of the SQLite database are detailed in Tables 2–4.

**Table 2:** Schema overview of Articles table.

Column name	Description
id	Unique identifier for each article (UUID format).
url	Original URL of the article.
tags	Tags associated with the article.
categories	Categories associated with the article.
hostname	Hostname of the article’s source.
date	Publication date of the article in ISO format (YYYY-MM-DD).
date_crawled	Date when the article was crawled in ISO format (YYYY-MM-DD HH:MM).

**Table 3:** Schema overview of Locations table.

Column name	Description
location_id	Unique identifier for each location.
loc_normal	Normalized name of the location (for geocoding purpose).
latitude	Latitude coordinate of the location.
longitude	Longitude coordinate of the location.
NUTS	Nomenclature of Territorial Units for Statistics identifier.
GEN	General Name of the NUTS location
ARS	Regional identification number

**Table 4:** Schema overview of Article_Locations table.

Column name	Description
article_id	Unique identifier of the article (foreign key to Articles.id).
location_id	Unique identifier of the location (foreign key to Locations.location_id).

In the dataset, 97.5% of the articles include an excerpt, providing readers with a brief summary to help them quickly grasp the main points. Nearly all articles (99.9%) come with a title, offering a clear initial indication of the article’s subject matter and drawing readers in. The categories and tags fields in the SQLite database are extracted directly from the raw HTML metadata of the original webpages. These fields reflect the source-specific metadata provided by each news outlet and are not harmonized across different sources. As such, values in the categories field may vary in granularity and naming conventions depending on the structure and editorial taxonomy of the original publisher. Both categories and tags are stored as plain text strings in the SQLite database, with multiple entries separated by commas. 65,3% of all articles include tags, while 35.5% include categories. The dataset contains 11,622,428 unique tag values and 462,679 unique category values. Users of the dataset should be aware of this variability and may consider additional preprocessing or mapping strategies to harmonize the values if needed for downstream tasks.

### SQLite database

The Articles table contains information about all articles, including their unique identifiers, URLs, and metadata. The schema of the table is as follows:

The Locations table contains information about geographic locations, including normalized names and geographic coordinates. The schema of the table is as follows:

The Article_Locations table serves as a join table, linking articles from the Articles table to geographic locations in the Locations table. This table supports a many-to-many relationship between articles and locations. The schema of the table is as follows:

The Article_Vectors table serves as a bridge between the vector store and the SQLite database. In the vector store, the IDs correspond to hashed article_id values. The table schema is outlined in Table 5.

**Table 5:** Schema overview of Article_Vectors table.

Column name	Description
article_id	Unique identifier of the article (foreign key to Articles.id).
hashed_id	Hashed version of the article_id used in the vector store

### Usearch vector database

**Fig. 6:**
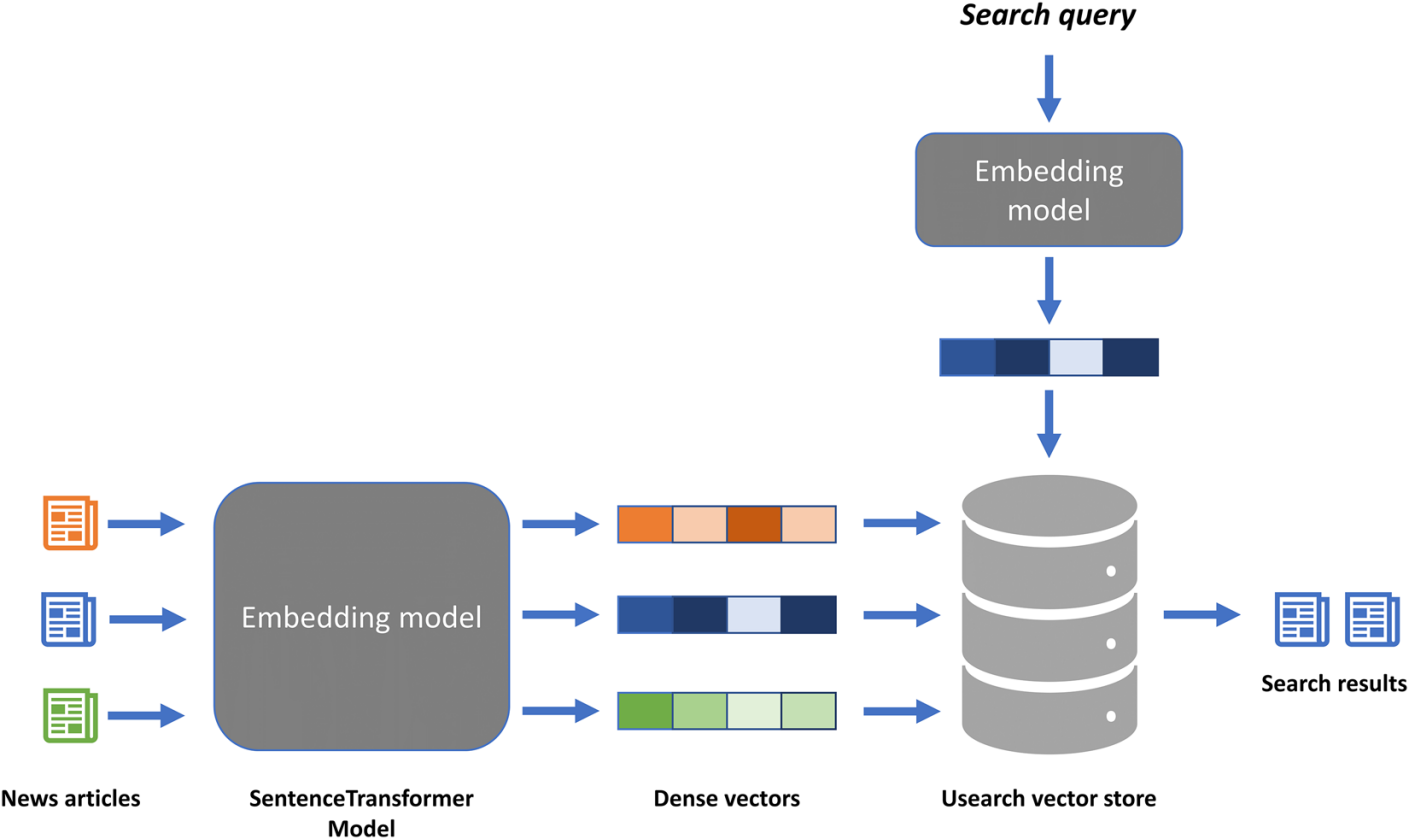
Vector database architecture (visual based on^41^).

The Usearch^37^ vector database enhances the dataset by enabling semantic search through vector representations of the articles. Each article in the SQLite database is associated with a high-dimensional vector in the vector database, capturing the semantic content of the article. This structure facilitates efficient similarity-based retrieval. To locate articles related to a specific topic or keyword, the keyword is transformed into a numerical query vector, which is then compared against the stored article vectors to measure similarity and identify relevant results. Figure 6 depicts the architecture of the vector database.

We provide example code for querying and filtering the database semantically as well as model recommendations. Moreover, we publish the vector data in different levels of granularity to meet diverse performance and storage requirements. Table 6 details the different versions of the vector database, leveraging quantization techniques to optimize for size, speed, and accuracy trade-offs.

**Table 6:** Overview of vector database quantizations.

Name	Quantization	Size	Distance metric
NewsIndex_f32	Float32	215 GB	Cosine distance
NewsIndex_int8	Int8	60GB	Inner product
NewsIndex_binary	Binary	14 GB	Hamming distance

Embedding quantization is a method to reduce storage requirements and computational costs while maintaining sufficient accuracy for semantic search. Quantization techniques, such as reducing embeddings from 32-bit floating-point precision (Float32) to 8-bit integers (Int8) or binary representations, can reduce the size of the vector data significantly with some trade-off in retrieval precision^38^.

The integration of the vector database with the SQLite database enables advanced query capabilities. Users can perform semantic searches to find articles with similar content and then retrieve associated metadata and geographic information from the SQLite database. This combination facilitates a wide range of analyses, including geographic trends in article topics and content similarity analysis.

## Technical Validation

To ensure the robustness and reliability of our dataset and methods, we conducted a series of validations targeting key components of the data processing pipeline. These validations assess the quality and consistency of the named entity recognition, geocoding, and vector database functionality, ensuring they meet the standards required for subsequent analyses*.* The validation processes were designed to evaluate the spatial, semantic, and temporal accuracy of our approach, emphasizing the dataset’s ability to reflect real-world patterns and trends.

### Named entity recognition and geocoding

**Fig. 7:**
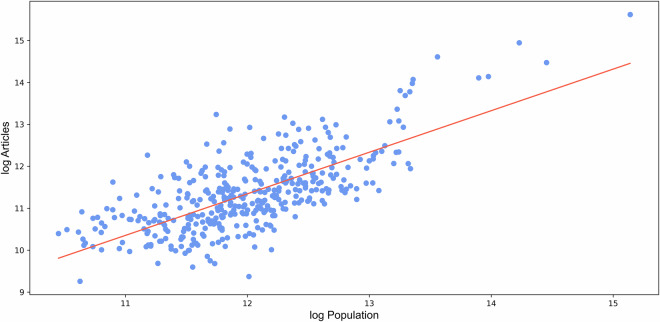
Number of articles and population size at NUTS-3 level.

In validating the named entity recognition (NER) and geocoding processes, we employed a log-log linear regression model. Figure 7 shows the relationship between number of articles and population size at NUTS-3 level. The analysis produced a coefficient of 0.99 (p < 0.001) for the relationship between population size (2022) and the pooled number of news articles, with an R² value of 0.507. This indicates that 50.7% of the variance in news article counts is explained by population size, confirming that our geocoding process effectively captures the association between population and news coverage at NUTS-3 level. The significant coefficient of 0.99 suggests that the number of news articles scales nearly proportionally with population size. The consistent linear relationship serves as evidence that our geocoding method is capable of correctly attributing news articles to the relevant locations, confirming that it is neither overestimating nor underestimating the distribution of news coverage relative to population. This ensures that our geocoding approach is reliable for subsequent analyses that depend on accurate spatial allocation of news data.

**Fig. 8:**
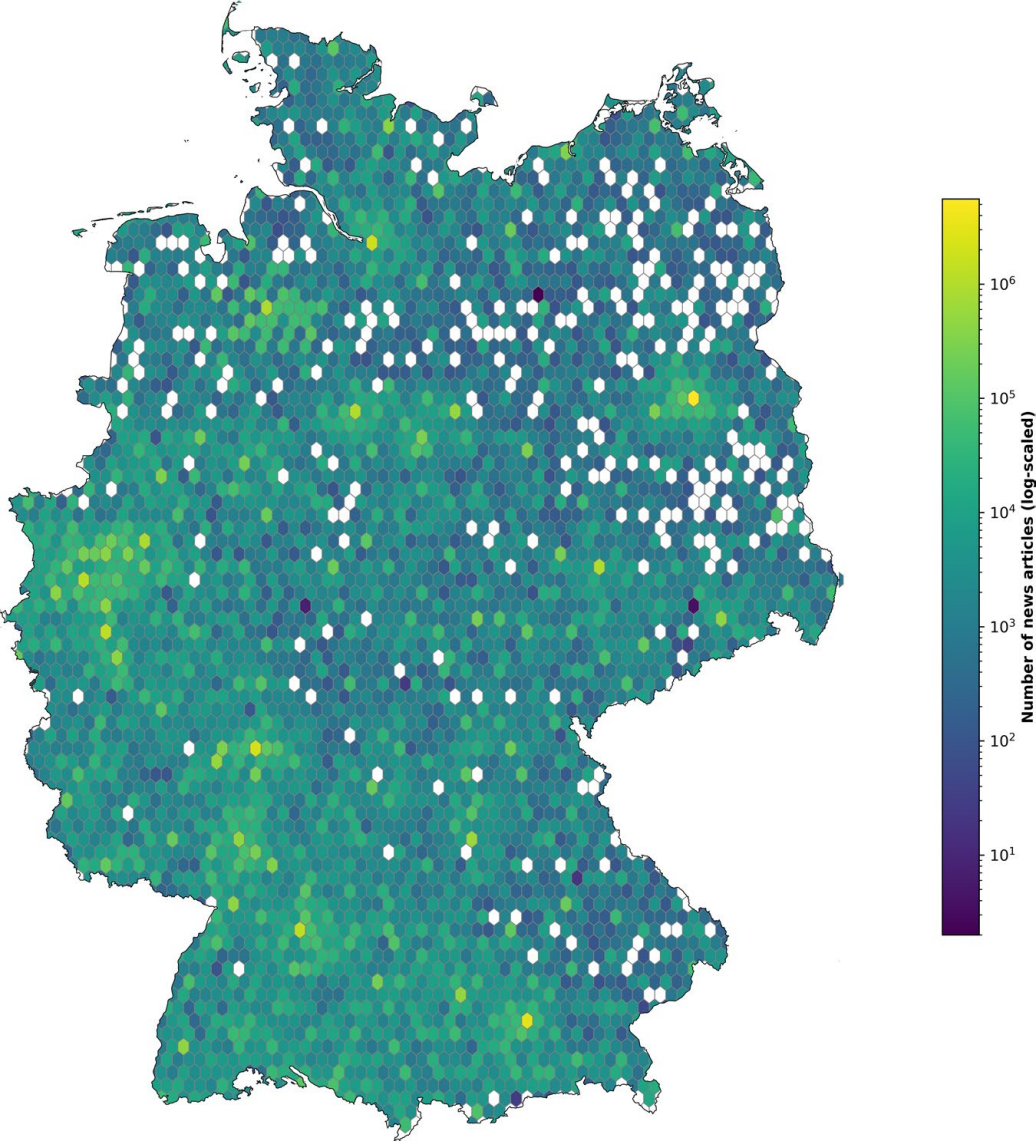
Number of news articles per location.

The map in Fig. 8 displays the number of news articles per location (log-scaled) using a hexbin density visualization. Each news article is assigned to a hexagon with an area of 44 km² based on the identified location(s). This type of visualization enables the assessment of count data on a map, illustrating the spatial distribution of news articles mentioning locations. It reveals spatial concentrations in the distribution of news mentions across Germany. Major cities such as Berlin, Hamburg, Munich, and Frankfurt dominate the news landscape, each being mentioned in a significant number of articles. One important observation from the map is that our dataset includes news articles associated with locations across the entire country, with only a few empty spots in sparsely populated areas of Germany. These empty hexagons predominantly cover forests, agricultural areas, or other unpopulated areas. When aggregating the location data to the NUTS3 level, the dataset includes news articles for all 400 NUTS3 regions. This comprehensive coverage enables regional analyses across the entire country, highlighting the dataset’s value for geographical research. The map also clearly shows that the number of news articles per location correlates positively with population size, as evidenced by the high number of articles for major cities.

**Fig. 9:**
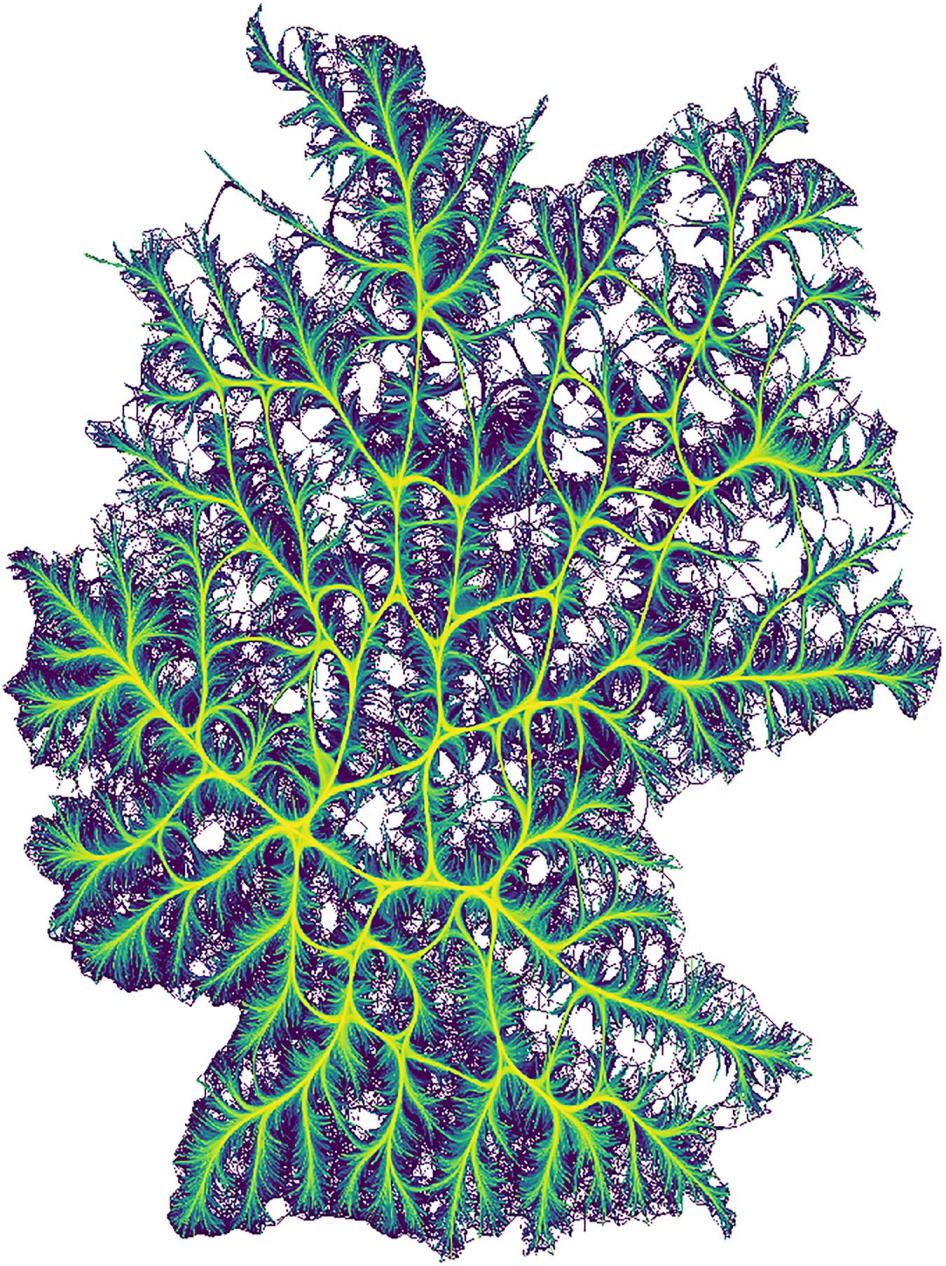
Network of co-occurrences of locations in articles.

As an additional validation of the geocoding pipeline, we analyzed relational patterns between German locations based on city name co-occurrences within individual news articles. The resulting network is depicted in Fig. 9. This co-occurrence network serves as a quality check, allowing us to assess whether the identified location entities and their resolved coordinates reflect plausible geographic relationships. Specifically, we constructed a network in which nodes represent cities and edges denote the number of articles in which two cities are mentioned together. The presence of strong and coherent links between major urban centers—as well as between cities and their surrounding regions—provides evidence that the underlying geoparsing has captured plausible spatial structures.

To validate the performance of our NER model, we conducted an evaluation using a randomly selected sample of 500 news articles, which were manually annotated for comparison. This manual annotation served as the ground truth against which the model’s performance was assessed. The evaluation yielded an entity-level F1-score of 93.87%, indicating a high level of accuracy in recognizing entities. The balance between recall and precision suggests that the model performs consistently in identifying entities without significant bias toward either false positives or false negatives. Results of the evaluation are detailed in Table 7, showcasing the precision, recall, and F1-score metrics that confirm the model performance.

**Table 7:** Performance metrics of NER model.

Precision	Recall	F1-Score
0.9363	0.9412	0.9387

### Vector database

To validate the functionality and effectiveness of the vector database, we conduct a use case focusing on “heat pumps.” A heat pump is a device that moves heat to warm or cool a building by extracting heat from the outside air or the ground and transferring it indoors. Heat pumps have garnered significant media attention in Germany in recent years, making them an informative and suitable example for this use case. Our aim is to demonstrate the precision of the semantic search capabilities and compare the extracted news volume against an independent data source—Google Trends—to ensure the robustness of the results. Using the vector database in 32-bit floating-point precision, we perform a semantic search for the term “heat pumps” in our German news dataset. By leveraging the pre-computed embeddings, the search algorithm retrieves articles whose semantic content closely aligns with the term, not just exact keyword matches. This allows us to capture articles that discuss heat pumps in various contexts, even when alternative phrasing or technical jargon is used.

We employ a two-step filtering process using the vector database to retrieve relevant articles. First, we use precomputed embeddings (Bi-encoder) to filter articles with a similarity score greater than 0.7. This initial step quickly narrows down the dataset by identifying articles semantically similar to “heat pumps” across various contexts, even when different terminologies are used.

Next, the remaining articles are fed into a more fine-grained reranker model^39^. This model performs a more precise evaluation, and we retain all articles with a similarity score greater than 0.1. This two-stage approach allows for a balance between computational efficiency and retrieval accuracy, ensuring that only relevant articles are selected for further analysis.

To evaluate the accuracy of different vector database configurations, we compare three precision levels: Binary, Int8, and Float32. We conduct an identical search across all three databases for the query “heat pumps”, retrieving the 300,000 nearest results in each case. A threshold of k = 300,000 corresponds to a cosine similarity of 0.7 in the Float32 database. The retrieved results from each database are subsequently processed through the same reranker model. Table 8 shows a comparison of retrieval accuracy and database size across the three precision levels, with the Float32 configuration serving as the benchmark.

**Table 8:** Comparison of retrieval accuracy across different precision levels.

Precision	Retrieval accuracy	Size
Float32	100%	215 GB
Int8	93.55%	60 GB
Binary	51.14%	14 GB

Our results demonstrate that the Int8 precision offers an interesting balance between size and accuracy, achieving a performance retention of 93.55% while offering a 4x reduction in size compared to the Float32 configuration.

To externally validate the results, we compare the temporal distribution of heat pump-related news articles from our dataset against data from the Google Trends News Index for the same term in Germany over the corresponding time period. Google Trends provides a normalized measure of search interest, allowing us to benchmark the frequency of media coverage against public interest. Similarly, we normalized the Common Crawl news data by scaling article counts into percentages relative to the highest observed count, facilitating a comparative analysis of media coverage trends.

**Fig. 10:**
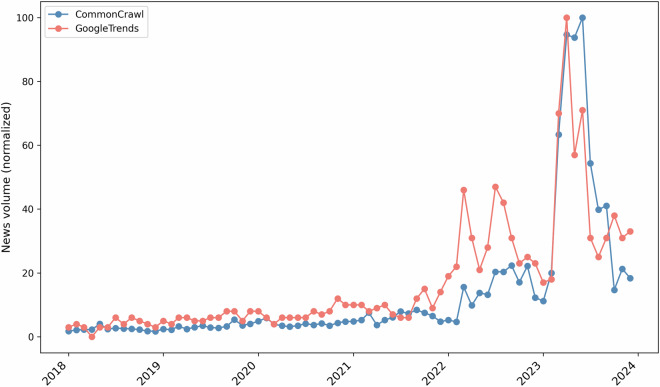
Comparison of news volumes.

Figure 10 illustrates the comparison between the volume of “heat pumps” articles and Google Trends data from 2018 to 2024. The two datasets show a strong correlation, with peaks in media coverage closely mirroring peaks in Google search interest. This alignment is particularly evident in 2023, when the energy crisis and governmental debates surrounding the Buildings Energy Act significantly heightened public interest in renewable energy technologies^40^.

The Pearson correlation coefficient between the monthly news article count and Google Trends data is 0.84, demonstrating a strong linear relationship. In sum, the use case highlights the reliability of the dataset and confirms the effectiveness of the filtering process.

## Usage Notes

The dataset can be linked to other spatial data through the geographic information provided, such as the NUTS identifier. The dataset contains a large compilation of news articles metadata, many of which may not be relevant to every use case. In addition to utilizing semantic search via the vector database to produce relevant subsets of the news data tailored to a specific use case, users may also consider filtering the database by other available variables. For example, users might subset by news provider, location, or tag, among other possibilities. This will facilitate data handling. Since the dataset is based on Common Crawl News data, it does not include news articles behind paywalls. Article titles and texts can be retrieved directly from Common Crawl and linked to this dataset using the provided IDs.

## Data Availability

All Python code produced for this project can be accessed on: https://github.com/LukasKriesch/CommonCrawlNewsDataSet.
